# Anti-Adhesive Properties of Calcium Alginate from *Sargassum fusiforme* against Particulate Matter-Induced Inflammation

**DOI:** 10.3390/cimb44020043

**Published:** 2022-01-25

**Authors:** Yun-Hua Fu, Xing-Yu Tao, Di Yang, Xue Li, Dong-Yue Zhou, Yu-Lin Dai, You-Jin Jeon

**Affiliations:** 1 Key Laboratory of Active Substances and Biological Mechanisms of Ginseng Efficacy, Ministry of Education, Jilin Ginseng Academy, Changchun University of Chinese Medicine, Changchun 130117, China; fuyh@ccucm.edu.cn (Y.-H.F.); taoxy@ccucm.edu.cn (X.-Y.T.); yangd@ccucm.edu.cn (D.Y.); lix@ccucm.edu.cn (X.L.); zhoudy@ccucm.edu.cn (D.-Y.Z.); 2 Department of Marine Life Science, Jeju National University, Jeju 63243, Korea; 3 Marine Science Institute, Jeju National University, Jeju 63333, Korea

**Keywords:** anti-adhesive, calcium alginate, particulate matter, *Sargassum fusiforme*

## Abstract

Fine dust generated by particulate matter (PM) pollution is a serious ecological issue in industrialized countries and causes disorders of the respiratory system and skin in humans. In the previous study, *Sargassum fusiforme* was treated with citric acid to remove heavy metals. In this study, the transfer of PM-mediated inflammatory responses through the skin to macrophages was evaluated. Moreover, the anti-adhesive effects of calcium alginate isolated from *S. fusiforme* (SFCA) against PM-induced inflammation were investigated. The structures of processing and unprocessing SFCA were then analyzed by Fourier-transform infrared spectroscopy (FT-IR), revealing minimal change after acid-processing. SFCA had protective effects both in PM-stimulated HaCaT keratinocytes and RAW 264.7 macrophages. In cellular environments, it was found that SFCA attenuated signal protein expressions such as inducible nitric oxide synthase (iNOS), cyclooxygenase (COX)-2, prostaglandin E_2_ (PGE_2_), and pro-inflammatory cytokines. Furthermore, macrophages were added to the culture medium of PM-stimulated keratinocytes to induce inflammation. SFCA was observed to significantly inhibit inflammatory responses; additionally, SFCA showed an in vivo anti-adhesive effect in zebrafish embryos.

## 1. Introduction

Air contaminants have become an increasingly concerning ecological issue, particularly in developed countries. Particulate matter (PM) particles are harmful substances in the air and have detrimental effects on many organisms [1]. Over the last two decades, China’s dramatic economic growth has been accompanied by reoccurring haze and smog episodes due to overusing coal-fired power production, vehicles, and agricultural activities [2]. PM with potentially toxic aerosolized metals is often present in Asia at concentrations well above natural environmental conditions [3,4]. This is associated with a high-risk of diseases including lung cancer, chronic respiratory and heart disease, weakening of the immune system, and decreases in lung function [5]. These particles have been proven to worsen respiratory issues and elicit allergic inflammatory responses in cells [6,7]. Exposure to air pollution, particularly in highly exposed regions such as the face, neck, and arms, could cause alterations in growth and differentiation patterns of exposed tissues. This, in turn, may result in inflammation or immune suppression that compromise the appearance of skin [8]. The evaluation of agents to counter PM-induced inflammation in human skin is, therefore, essential.

Brown seaweed *Sargassum fusiforme* has been used as a traditional medicine ingredient in oriental countries [9]. Calcium alginate from seaweed is a protonated water-insoluble molecule comprising straight binary polymers [10,11]. They form hydrogels, chelate metals, and may possess various other properties of interest [12]. Due to high contents of inorganic arsenic (iAs) found in marine algae compared to other foods [13], our previous study decreased heavy metal concentrations in *S. fusiforme* by acid washing [14]. In this study, the anti-adhesive and protective effect of calcium alginate from *S. fusiforme* against PM-induced inflammatory responses was evaluated.

## 2. Materials and Methods

### 2.1. Materials

PM (CRM No. 28) was obtained from the National Institute for Environmental Studies (Ibaraki, Japan). Morphological and compositional analyses of PM were demonstrated in our previous study [15]. Heavy metal salts, potassium bromide, potassium permanganate, hydrogen peroxide, and nitric acid were purchased from Sigma-Aldrich (St Louis, MO, USA). Commercial sodium alginate was purchased from DaeJung chemicals metals Co., Ltd. (Siheung-si, Korea). Multi-element calibration standard for inductively coupled plasma optical emission spectroscopy (ICP-OES) was purchased from Perkin Elmer Inc. (Norwalk, CT, USA). ELISA kits were obtained from Thermo Fisher Scientific (Waltham, MA, USA) and Invitrogen (Carlsbad, CA, USA). Other chemicals were obtained from commercial and were of high analytical grade.

### 2.2. Extraction of Calcium Alginate

*S. fusiforme* was harvested from Jeju, Korea, in May 2019, and the collection position is 33°25′24.1″ N/126°55′22.7″ E. The repository was deposited in Jeju National University (Jeju, Korea). *S. fusiforme* was pretreated with acid-washing to decrease heavy mental content. The method of calcium alginate extraction was proceeded according to the previous study [16]. Briefly, *S. fusiforme* powder was de-pigmented with 95% ethanol and soaked in 10% formaldehyde overnight. After filtering, the solution of 95% ethanol was used to replace any formaldehyde. Freeze-dried powder was soaked in distilled water containing HCl and maintained at a pH of 4.0. The suspension was stirred by magnetic bar at room temperature for 24 h and then filtered and washed with distilled water. Distilled water containing 5% Na_2_CO_3_ (*w*/*v*) was then used to extract calcium alginate. The extract was filtered and centrifuged (10,000× *g* for 10 min, 4 °C). The supernatant was collected and the pH value was increased to 6.0 by using HCl. Subsequently, calcium alginic acid was precipitated when treated with excess CaCl_2_ solution. Sodium alginate and calcium alginate were centrifuged and dissolved in 10% HCl, and centrifugation was performed again for collection. After at least six cycles of washing, the precipitate was redissolved and pH was neutralized at 7.0. Dialyzed and lyophilized powder was considered as calcium alginate from *S. fusiforme* (SFCA).

### 2.3. Analysis of Composition and Heavy Metal Content

The proximate composition of SFCA was determined by the following methods. Metal content was analyzed according the standard method. The muffle furnace was employed to analyze ash content with the temperature at 600 °C for 6 h [17]. The protein content of SFCA was measured using the Kjeldahl method with bovine serum albumin as the standard; the total carbohydrate content of SFCA was measured by the phenol sulfuric acid method using glucose as the standard; the Folin–Ciocalteu method was applied to analyze polyphenol content with gallic acid as the standard [18].

SFCAs were analyzed using a Nicolet 6700 FT-IR spectrometer (Thermo Fisher Scientific, MA, USA). The frequency range of 500–4000 cm^−1^ was used for spectral measurements. Proton nuclear magnetic resonance (^1^H NMR) spectroscopy was acquired on SFCA solution (1% *w*/*v*) in D_2_O, with recordings at 80 °C by using an AVANCE III HD 400 (Bruker Scientific Instruments, Billerica, MA, USA) spectrometer operating at a frequency of 400 MHz. The mannuronic acid to guluronic acid (M/G) ratio was analyzed according to the previous method [19].

### 2.4. Cell Culture

RAW 264.7 macrophages and HaCaT keratinocytes were purchased from American Type Culture Collection and cultured at 37 °C in 5% CO_2_ incubator. Dulbecco’s Modified Eagle’s Medium (DMEM) maintained with 10% (*v*/*v*) fetal bovine serum (FBS) and 1% (*v*/*v*) penicillin and streptomycin was obtained from Thermo Fisher Scientific (Waltham, MA, USA).

### 2.5. Measurement of Cell Viability, ROS Production, and Nuclear Staining

Cells (1 × 10^5^ cells mL^−1^) were seeded in 96-well culture plates. The increasing concentrations (25, 50 and 100 μg mL^−1^) of SFCA were treated in the presence or absence of wells. After 1 h incubation, cells were substituted in fresh medium before treating PM (125 μg mL^−1^) for 30 min incubation. Cells untreated with SFCA and PM were considered as control. Further incubation for 24 h was performed in 50 μL phosphate-buffered saline (PBS) containing 2 mg mL^−1^ of 3-(4,5-dimethylthiazol-2-yl)-2, 5-diphenyltetrazolium bromide (MTT) for the detection of cell death. Cells were incubated for 3 h in the dark, and then 200 μL of dimethyl sulfoxide (DMSO) was replaced by an excess medium in each well. The DMSO solution containing formazan was centrifuged (10,000× *g* for 5 min) and the supernatant was measured at a wavelength of 540 nm. For ROS production, after 24 h incubation with samples, cells were treated in 10 μL PBS containing 5 μg mL^−1^ of 2, 7-dichlorofluorescein diacetate (DCFH-DA) and incubated for 10 min in the dark. ROS productions of cells were detected using a fluorescence microscope equipped with a CoolSNAP-Pro color digital camera (Meyer Instruments, Inc., Houston, TX, USA).

The protective properties of SFCA were identified on nuclear morphology changes in PM-induced keratinocytes following the protocol of Hoechst 33,342 staining and DCFH-DA assay. Keratinocytes were seeded (1 × 10^5^ cells mL^−1^) in 24-well culture plates. Various concentrations SFCA were treated in the presence or absence to cells with 24 h incubation. After 24 h incubation, Hoechst 33,342 (stock 1 mg mL^−1^) and DCFH-DA solution (5 µg mL^−1^) were added to each well. Cells were stained in 10 min in the dark and imaged by a fluorescence microscope (equipment is the same as ROS measurement).

### 2.6. Inflammatory Responses and Enzyme Immunoassay Measurement

RAW 264.7 macrophages and HaCaT keratinocytes were pre-seeded separately in different 24-well plates with 24 h incubation. The increasing concentrations of SFCA were treated to each well. Following incubation for 1 h, cells were added to the presence or absence of PM at a concentration of 125 µg mL^−1^. The cells were substituted in fresh medium after additional 30-minnute incubation. Culture plates were continuously incubated for 24 h. The medium from cell was collected for measurement of the inflammatory-related mediators. Cell viability, intracellular ROS, metal ions contents, inflammatory-related mediators, and cytokines were evaluated using MTT assay, DCFH-DA assay [19], ICP-OES assay [20], and commercial kit.

Pre-seeded keratinocytes were treated with different concentrations of SFCA, and inflammation was induced by PM for 30 min. The wells were replenished with fresh DMEM and incubated for 24 h. The culture medium of PM-induced keratinocytes (H-PM) were treated with pre-seeded macrophages in real time and analyzed for all inflammatory-related mediators after 24 h incubation.

The expressions of mediators and cytokines in cell medium were determined by commercial ELISA kits. The analysis method followed the manufacturer’s guidelines.

### 2.7. Western Blot Assay

Cells were collected after SFCA and stimulator (PM or H-PM) treatment by step. A lysis buffer was used to homogenized cells. Lysates were centrifuged, and the supernatant was collected. Protein content measurement of the supernatant was carried out by using a commercial BCA kit. Polyacrylamide gel (10%) was used to separate lysis buffer containing 20 μg protein. The protein on polyacrylamide gel was transferred onto a nitrocellulose membrane. The membrane was blocked by blocking buffer and incubated with primary antibodies (anti-inducible nitric oxide synthase (iNOS) and anti-cyclooxygenase (COX)-2) and secondary antibodies. The antibodies were obtained from Santa Cruz Biotechnology (Dallas, TX, USA). Chemiluminescent substrate was used to develop blots, and fluorescence images were performed to FUSION SOLO Vilber Lourmat system (FUSION, Paris, France) [21].

### 2.8. Zebrafish Embryo Assay

Adult zebrafishes were obtained from Nanjing EzeRinka Biotechnology Co., Ltd. (Nanjing, China), and embryos were harvested by natural spawning with a 14/10 h light/dark cycle in 28.5 °C. The experiments of PM-induced cell death, ROS, and NO production in embryos were evaluated. The treated concentration of PM at 10 μg mL^−1^ was used in embryo viability assay. In brief, the embryos were divided to culture plates randomly and added to different concentrations SFCA followed by PM treatment. Following incubation for 72 h, hatched larvae were stained by acridine orange, 4-Amino-5-methylamino-2′, 7′-difluorofluorescein diacetate (DAF-FM DA), and DCFH-DA in the dark, respectively. Those were used for the determination of cell death, NO production, and ROS production in larvae. The fluorescence intensity of larvae was developed by spectrofluorometer (Perkin–Elmer LS-5B, Wien, Austria).

### 2.9. Statistical Analysis

All assays were performed in three independent experiments. Values were expressed as the mean ± standard error (SE). Mean values in GraphPad prism 5 were analyzed by one-way ANOVA. Means of the parameters were analyzed by Student’s *t*-test. * *p* < 0.05 and ** *p* < 0.01 were considered as significant differences.

## 3. Results

### 3.1. Chemical and Structural Features of SFCA

As shown in Table 1, SFCA is rich in carbohydrate content, whereas it has relatively lower ash content due to the removal of minerals during processing. SFCA was lower in the amounts of proteins and polyphenols. This approximate chemical composition implies SFCA is well prepared with higher purity. SFCA was characterized by proton nuclear magnetic resonance spectroscopy, and the resulting M/G ratio was 1.25.

**Figure 1:**
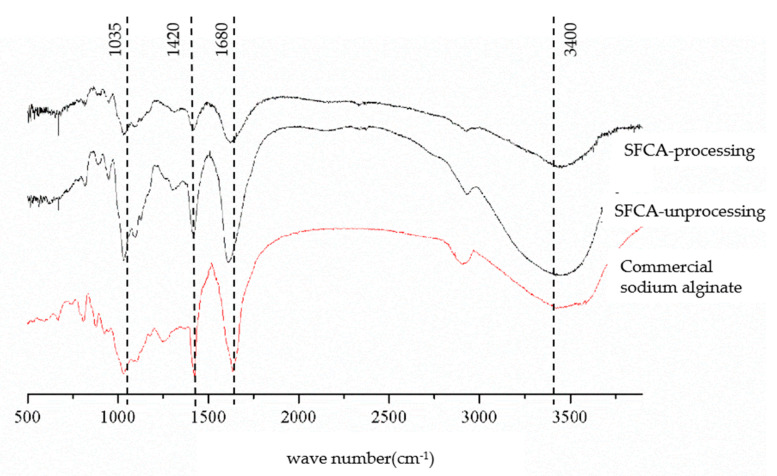
FT-IR spectroscopic analysis of structural features of SFCA. SFCA-processing: calcium alginate gels isolated from citric acid washed *S. fusiforme*; SFCA-unprocessing: calcium alginate gels isolated from original *S. fusiforme*.

In Figure 1, FT-IR spectrum peaks were found at wave numbers 3400 cm^−1^, 1680 cm^−1^, 1420 cm^−1^, and 1035 cm^−1^ in all test samples. These peaks in unprocessing SFCA were stronger than in acid-processing spectra, indicating acid-processing influences on the structure of SFCA. Molecular chains with unstable structure of polymer were affected by acid-processing.

### 3.2. Compositional Analysis of PM-Stimulated Keratinocytes with/without SFCA Treatment

The optimal PM concentration and exposure time followed the previous study for cell viability and inflammation induction [22]. After PM and SFCA treatment for 24 h, keratinocytes were recovered and analyzed for the composition of metal ions by ICP-OES. Relatively stable concentrations of K and Na and significant decreases such as Mg, Ca, Fe, Cu, Sr, Ba, and Pb were found in the comparison with PM only treated cells (Table 2). The reduction in the metal ions was observed with increasing concentrations of SFCA. This implies SFCA could well reduce harmful metals existing in PM and protects human skin.

### 3.3. Protective Effect of SFCA against PM-Induced Inflammation in Keratinocytes

**Figure 2:**
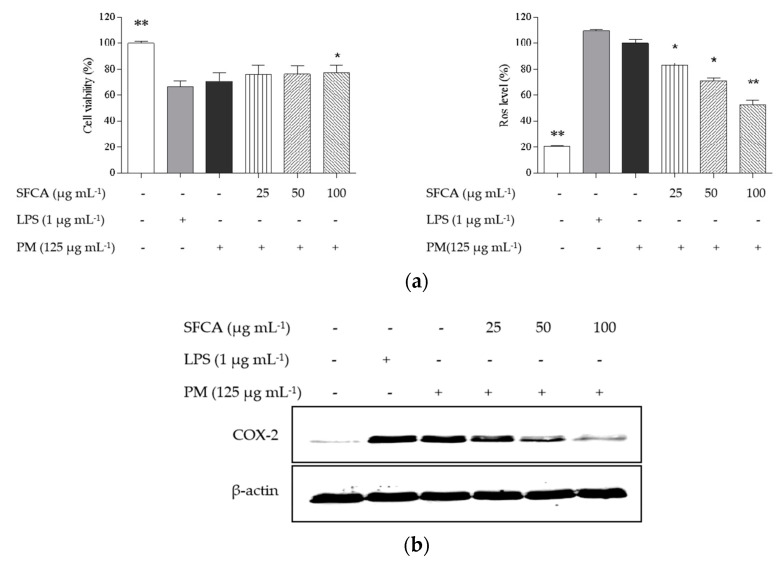

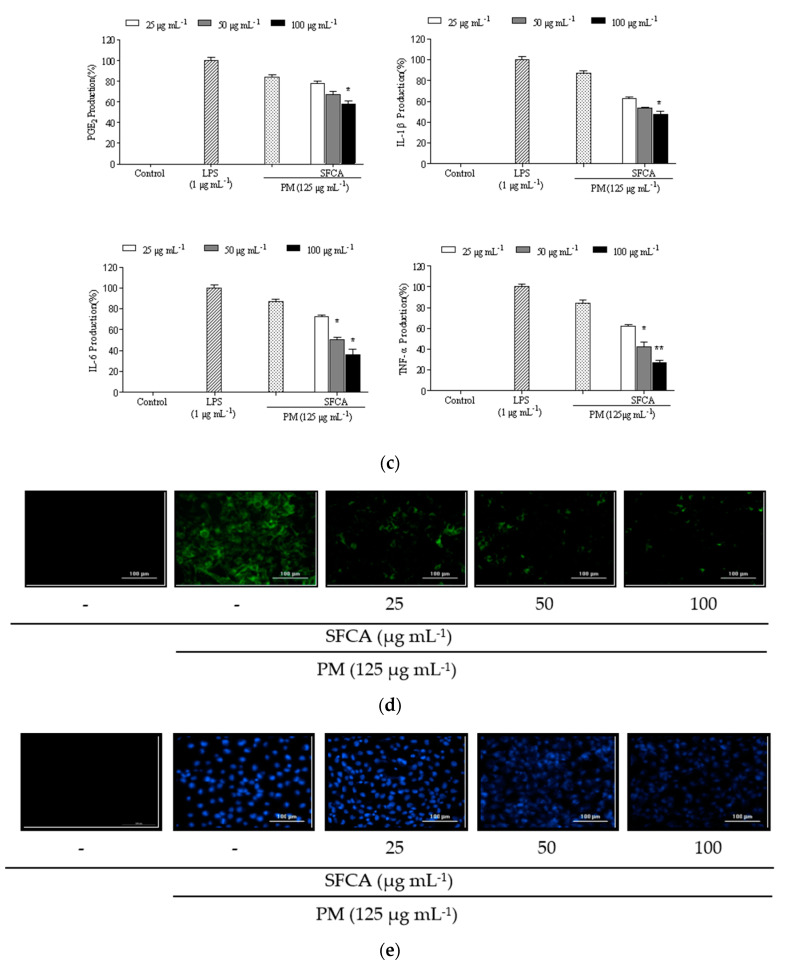
Protective effect of SFCA against inflammation induced by PM in HaCaT keratinocytes. (**a**) Analyses of HaCaT cell viability and intracellular ROS levels; (**b**) Western blot analyses of COX-2 expressions; and (**c**) ELISA of prostaglandin E_2_ (PGE_2_) and pro-inflammatory cytokines (IL-1β, IL-6, and TNF-α). Pre-seeded cells (1 × 10^5^ cells mL^−1^) were treated with or without different SFCA concentrations during 24 h and stimulated with PM after 30 min. Cells were harvested after 24 h to measure inflammatory mediators (COX-2 and PGE_2_) and pro-inflammatory cytokines (IL-1β, IL-6, and TNF-α). Apoptotic body formation was observed under a fluorescence microscope after (**d**) DCFH-DA treatment and (**e**) Hoechst 33,342 staining. Graphical representations are means ± SE based on three replications. * *p* < 0.05 and ** *p* < 0.01 indicate that values were significantly different from those for the PM-treated group.

It is well known that lipopolysaccharide (LPS) induces inflammation as well as oxidation in HaCaT keratinocytes and then resulted in cell apoptosis and necrosis. In Figure 2, it was found that PM induced reduced cell viability as well as inflammation via expressed COX-2, PGE_2_ and IL-1β and oxidation via increased ROS level. SFCA protected PM-treated keratinocytes indicating increased cell viability at the highest concentration (Figure 2a). iNOS and NO are not produced in keratinocytes; therefore, other mediators including COX-2, tumor necrosis factor-α (TNF-α), and inflammatory cytokines were determined. As shown in Figure 2b,c, all mediators indicating PM-induced inflammation dose-dependently were decreased and significantly reduced at relatively higher concentrations. SFCA treatment substantially reversed PM-stimulated inflammatory responses in HaCaT keratinocytes. This result suggests that SFCA might have anti-adhesive effects for human skin.

### 3.4. Protective Effect of SFCA against PM-Induced Apoptotic Body Formation in Keratinocytes

Measurements of cell viability, ROS detection, and staining assay were followed by cell viability assay to further confirm the protective effect of SFCA against PM-stimulated keratinocytes. In Figure 2d,e, apoptotic body formations were observed in PM-stimulated cells using fluorescence microscopy. However, the number of the observed apoptotic bodies decreased with SFCA and the effects are dose-dependent.

### 3.5. Protective Effect of SFCA in PM-Stimulated Macrophages

**Figure 3:**
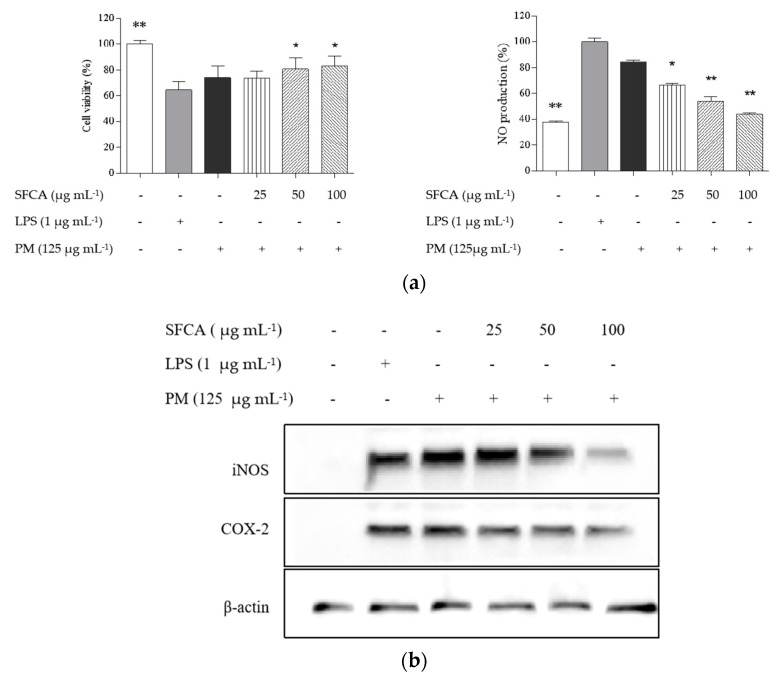

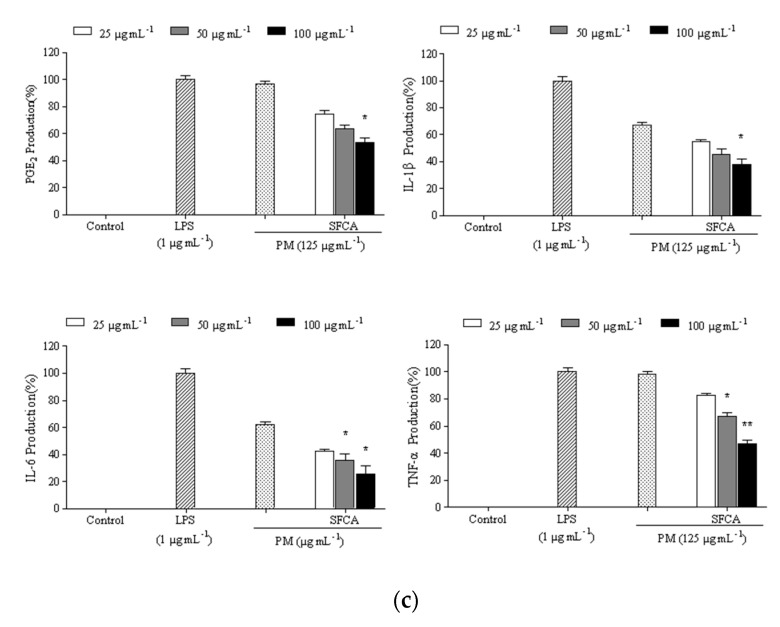
Efficacy of SFCA against inflammation induced by PM in RAW 264.7 macrophages (**a**), and analyses of RAW cell viability and intracellular ROS levels; (**b**) Western blot analyses of iNOS and COX-2 expressions; and (**c**) ELISA of PGE_2_ and pro-inflammatory cytokines (IL-1β, IL-6, and TNF-α). Pre-seeded cells (1 × 10^5^ cells mL^−1^) were treated with or without different SFCA concentrations after 24 h and stimulated with PM after 30 min. Cells were harvested after 24 h to measure inflammatory mediators (COX-2 and PGE_2_) and pro-inflammatory cytokines (IL-1β, IL-6, and TNF-α). Graphical representations are means ± SE based on three replications. * *p* < 0.05 and ** *p* < 0.01 indicate that values were significantly different from those for the PM-treated group.

In Figure 3a, NO production was upregulated in both PM and LPS-stimulated macrophages compared with untreated cells. Among these, NO production induced by PM was lower than that by LPS. In the SFCA treatments on PM-stimulated macrophages, NO productions decreased and cell viabilities increased in a dose-dependent manner. iNOS and COX-2 were regarded as key regulators in inflammatory field, which is strongly associated with the productions of NO and PGE_2_. Western blot analysis indicated that PM significantly upregulated the expressions of key inflammatory regulators such as iNOS and COX-2, whereas SFCA dose-dependently decreased with concentrations (Figure 3b). Consistently with these observations, SFCA lowered the contents of PGE_2_, TNF-α, IL-1β, and IL-6 overexpressed by PM stimulation, and significant decreases were observed at the highest concentration (Figure 3c). The results in the present study are in good agreement with those of previous studies [21].

### 3.6. Inflammatory Responses in Macrophages Induced with H-PM and SFCA

**Figure 4:**
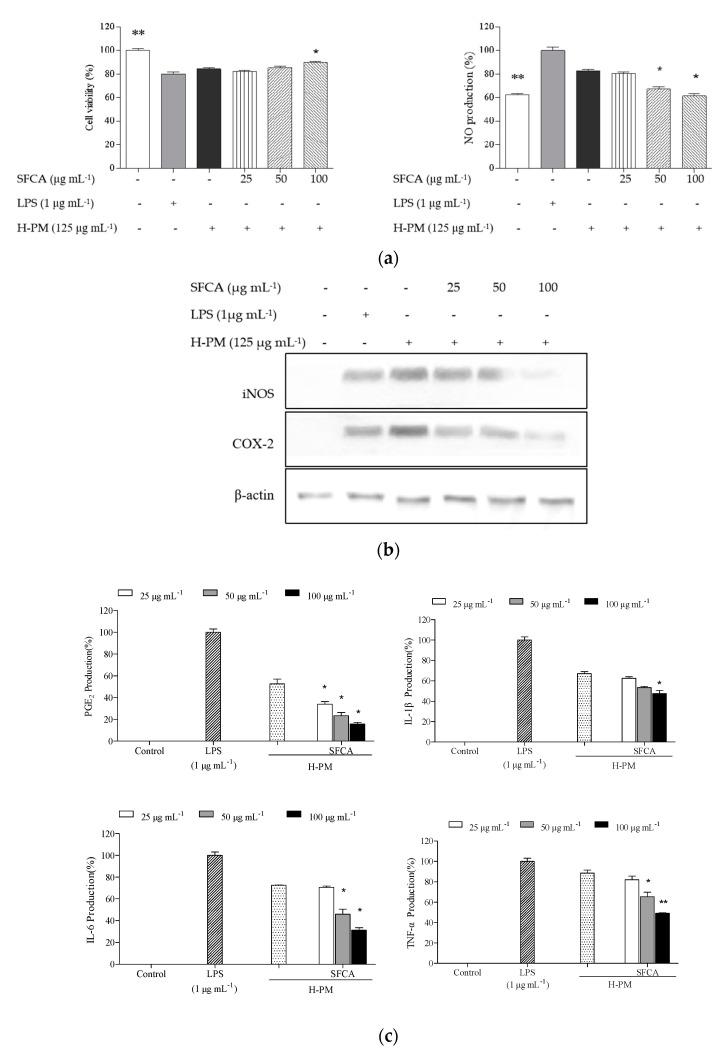
Inflammatory stimulation of the RAW 264.7 macrophages by the culture medium of PM-induced HaCaT cells and the anti-inflammatory effects of SFCA: NO production and cytotoxicity (**a**) and analysis of iNOS and COX-2 levels (**b**) and inflammatory mediators (**c**), including tumor necrosis factor α (TNF-α), interleukin (IL)-1β, IL-6, and PGE_2_. HaCaT cells were pre-seeded in culture plates 1 × 10^5^ cells mL^−1^), incubated for 24 h, and treated with or without different concentrations of SFCA. After 1 h, the cells were treated with PM (125 µg mL^−1^) and 24 h later, culture media were treated to each pre-seeded RAW 264.7 macrophage culture well plates in real time. The evaluations were conducted after a 24 h. Experiments were carried out in triplicate, and the results are represented as means ± SE. Values are significantly different from the positive control (PM treated group) at * *p* < 0.05 and ** *p* < 0.001. H-PM: cultured medium of PM-stimulated in keratinocytes.

The relationship between keratinocytes and macrophages in transferring inflammatory potential was investigated. Cell viability and NO production as well as expressions of iNOS, COX-2, PGE_2_, and pro-inflammatory cytokines were analyzed in H-PM-induced macrophages. SFCA treatment showed anti-adhesive effect and effectively decreased the inflammatory responses dose-dependently (Figure 4a). Accordingly, with an increase in SFCA concentrations, the intensities of iNOS and COX-2 decreased (Figure 4b). In Figure 4c, H-PM triggered an increase in inflammatory mediators and pro-inflammatory cytokines in macrophages compared to the blank. However, SFCA at relatively higher concentrations significantly lowered all expressions. In fact, IL-1β and IL-6 produced in keratinocytes stimulating inflammatory responses in macrophages were found [23]. Hence, PM-stimulated HaCaT keratinocytes contribute inflammatory mediators and pro-inflammatory cytokines that may trigger inflammatory responses in macrophages.

### 3.7. Protective Effect of SFCA in the PM-Stimulated In Vivo Zebrafish Model

**Figure 5:**
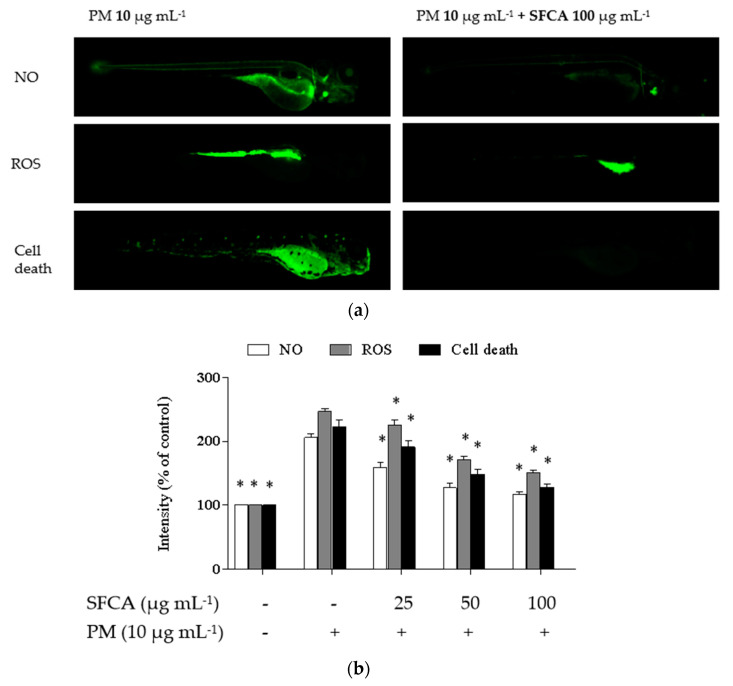
Inflammatory stimulation of zebrafish larvae by PM and anti-inflammatory effects of SFCA: (**a**) anti-inflammatory properties were evaluated by measuring NO and ROS production, and cell death in the zebrafish embryo model and (**b**) their intensities. Experiments were carried out in triplicate, and the results are represented as means ± SE. Values are significantly different from the positive control (PM treated group) at * *p* < 0.05.

As shown in Figure 5a, PM induced ROS and NO production as well as cell death but SFCA dose dependently reduced in zebrafish embryos. Especially, the cell death rate of embryos was decreased when pretreated with SFCA prior to PM treatment. From the in vivo zebrafish model, SFCA effectively decreased oxidation and inflammation via lowering ROS and NO production, which induced decreases in cell death in PM-induced zebrafish (Figure 5b).

## 4. Discussion

Generally, there are two main factors contributing to air pollution: anthropogenic activities and natural events. Anthropogenic activities that release emissions to the atmosphere include industrial expansion [24], coal or plant burning [25], vehicle consumption [26], and mineral particles [27]. Most of the air contamination in natural events is characterized by visible changes, such as hazy weather and sand storms from the Gobi desert [28]. PM particles, especially PM 2.5 (particulate matter less than 2.5 μm in size), cause severe damage to respiratory and cardiovascular systems [29] and are the main contributors to air pollution in East Asia [30]. Although PM has become a serious problem in most developing countries, there is limited information on PM chemical composition and mobility trends. Some studies have investigated ambient ultra-fine particles during the fine dust season in Gwangju, Korea and Stuttgart, Germany [31,32]. Accordingly, polluted air in China from 2011 to 2013 has been found to contain particles composed of Ca^2+^, Mg^2+^, K^+^, Na^+^, Cl^−^, SO_2_, SO_4_^2−^, NO_3_^−^, and NH_4_^+^ ions [33]. Another study investigated markers OC2, EC1, and NO_3_^−^/SO_4_^2−^ ratio in PM 2.5 emissions in Xi’an in the winters of 2006, 2008, and 2010 [34]. The composition of the dust (Fe, Co, Mg, Al, Ca, Ni, Zn, Sb, V, Cr, As, Se, Cd, Mn, and Pb) was analyzed by inductively coupled plasma mass spectrometry [35]. An interesting study describes the pathway of PM-induced inflammation and oxidative stress in macrophages [36]. The evidence supports the theory that dust particles can activate IL-1β, IL-16, NF-κB, and COX-2 expression in human myeloid leukemia cells, indicating strong inflammatory responses [37]. Although reports showed adverse effects of PM on single organ systems, this study investigated transfer inflammatory responses between skin cells and macrophages. In our results, SFCA strongly reduced metal ions with increasing concentrations. Hence, this implies that SFCA could significantly reduce harmful metals exiting in air pollution.

The keratinocyte model is widely used in dermatological studies to investigate results from the outside layers of the skin. Keratinocytes protect the inner skin cells. Once stimulated by PM, apoptosis was triggered, which likely manifests as skin irritation and damage [38], which further produces secondary mediators that upregulate the expression of IL-1β and IL-6, resulting in an inflammatory response in HaCaT cells [39]. Macrophages regulate inflammation via phagocytosis and antigen presentation and result in the production of many mediators and cytokines [40]. Macrophage activation is an important strategy to prevent infection, and it is stimulated by cytokines such as interferon γ, IL-1β, and TNF-α or by certain bacterial extracellular components such as LPS and external chemicals [41]. SFCA isolated from acid-processed *S. fusiforme* was assessed by the in vitro transfer inflammatory response system. The results found that SFCA lowered the contents of PGE_2_, TNF-α, IL-1β, and IL-6 overexpressed by PM stimulation in both skin cells and macrophages. Therefore, the evidence of reductive ability of SFCA in PM-induced inflammation was found.

In this study, PM induced an inflammatory effect in keratinocytes. Furthermore, the stimulator is probably a pro-inflammatory cytokine that both acts on keratinocytes and transfers inflammatory responses to macrophages. Previous studies have demonstrated anti-inflammatory and protective effects of marine bioactive components against PM-induced inflammation. *S. horneri* extract has been proposed as a potential treatment for PM induced inflammatory stress via p38 and Nrf2/HO-1 expression [42]. Moreover, the yield and structure of alginic acid from *S. horneri* and acid-processing *S. fusiforme* are different because alginic acid differs depending on the species [43], extraction method [44], and location [45]. Therefore, the anti-adhesive effect of SFCA against PM-induced damage was first evaluated. The result of the animal experiment in this study indicated that responses induced by PM could be markedly countered by SFCA treatment.

## 5. Conclusions

SFCA, calcium alginate, isolated from acid-processed *S. fusiformis* exhibited strong anti-adhesive activities via promoting cell growth and reducing inflammatory factors in PM-treated macrophages and keratinocytes in vitro. Additionally, SFCA showed protective effects against PM-induced damages in vivo 72 hpf zebrafish models. SFCA could be a candidate agent in anti-adhesive effects and cosmeceutical formulations. Furthermore, the present study could extend the appropriate application of algae in the management of fine dust particles.

## Figures and Tables

**Table 1:** Chemical composition of SFCA.

Constituent	Composition
Carbohydrate	89.25 ± 0.64%
Ash	2.41 ± 0.27%
Polyphenol	0.96 ± 0.01%
Protein	0.41 ± 0.02%
M/G ratio	1.25

Results are given as the means ± standard error (SE) based on triplicate determinations.

**Table 2:** Changes of elemental metal compositions in PM-induced keratinocytes with SFCA during 24 h.

Element (ppm)	Control	PM	PM+SFCA	PM+SFCA	PM+SFCA
			(25 μg mL^−1^)	(50 μg mL^−1^)	(100 μg mL^−1^)
K	454 ± 32	451 ± 32	456 ± 24	435 ± 26	424 ± 38
Ca	149 ± 15 *	435 ± 21	345 ± 26	159 ± 38	62 ± 25 **
Na	612 ± 9	609 ± 29	623 ± 22	657 ± 34	665 ± 25
Mg	115 ± 9	165 ± 23	132 ± 21	65 ± 14	38 ± 10 **
Sr	5 ± 6 **	149 ± 19	94 ± 12 *	44 ± 18 *	18 ± 20 **
Fe	49 ± 9 **	258 ± 35	165 ± 11	140 ± 25 *	94 ± 33 **
Al	N.D.	79 ± 14	75 ± 4	62 ± 8	51 ± 14
As	N.D.	39 ± 16	42 ± 18	31 ± 11	19 ± 6
Mn	N.D.	65 ± 6	45 ± 15	32 ± 14	11 ± 9
Pb	N.D.	368 ± 15	217 ± 38	111 ± 15 *	39 ± 25 **
Cu	N.D.	32 ± 15	32 ± 7	11 ± 10	1 ± 3
Cr	N.D.	32 ± 11	25 ± 15	12 ± 24	N.D.
Ba	N.D.	101 ± 11	78 ± 28	14 ± 12	N.D.

Results are given as the means ± SE based on triplicate determinations. For a specific element, * *p* < 0.05 and ** *p* < 0.01 were considered significant compared to the PM-treated group. (N.D. stands for “not detected”).

## Data Availability

All data, models, and code generated or used during the study appear in the submitted article.
